# Altering Dynamics of Autonomic Processing Therapy (ADAPT) trial: a novel, targeted treatment for reducing anxiety in joint hypermobility

**DOI:** 10.1186/s13063-021-05555-4

**Published:** 2021-09-21

**Authors:** Geoff Davies, Jenny L. L. Csecs, Heather Ball, Jess Dare, Stephen Bremner, Robin Hosking, Hugo D. Critchley, Nick Grey, Jessica A. Eccles

**Affiliations:** 1 Department of Neuroscience, Brighton and Sussex Medical School, University of Sussex, Falmer, UK; 2 Sussex Partnership NHS Foundation Trust, Worthing, UK; 3 Sussex Community NHS Foundation Trust, Brighton, UK; 4 Brighton and Sussex Clinical Trials Unit, Brighton & Sussex Medical School, Falmer, UK; 5 School of Psychology, University of Sussex, Falmer, UK; 6 Trafford Centre for Medical Research, University of Sussex, Falmer, BN1 9RY UK

**Keywords:** Hypermobility, Anxiety, CBT, Interoception, RCT

## Abstract

**Background:**

Hypermobility is a poorly recognised and understood musculoskeletal disorder thought to affect around 20% of the population. Hypermobility is associated with reduced physiological and psychological functioning and quality of life and is a known risk factor for the development of an anxiety disorder. To date, no evidence-based, targeted treatment for anxiety in the context of hypermobility exists. The present intervention (ADAPT—Altering Dynamics of Autonomic Processing Therapy) is a novel therapy combining bio-behavioural training with cognitive approaches from clinical health psychology targeting the catastrophisation of internal sensations, with aim to improve autonomic trait prediction error.

**Method:**

Eighty individuals with diagnosed hypermobility will be recruited and the efficacy of ADAPT to treat anxiety will be compared to an Emotion-Focused Supportive Therapy (EFST) comparator therapy in a randomised controlled trial. The primary treatment target will be post therapy score on the Beck Anxiety Inventory, and secondary outcomes will also be considered in relation to interoception, depression, alexithymia, social and work adjustment, panic symptoms and dissociation. Due to COVID restrictions, the intervention will be moved to online delivery and qualitative assessment of treatment tolerance to online therapy will also be assessed.

**Discussion:**

Online delivery of an intervention targeting anxiety would improve the quality of life for those experiencing anxiety disorder and help to reduce the £11.7 billion that anxiety disorders cost the UK economy annually.

**Trial registration:**

World Health Organization ISRCTN17018615. Registered on 20th February 2019; trial protocol version 2

## Introduction

Hypermobility is a poorly-recognised and understood musculoskeletal disorder [1], affecting approximately 20% of the population [2]. Hypermobile individuals are more likely to experience severe chronic widespread pain and have co-morbid rheumatic conditions (60%) [3] and dysautonomia [4]. Generally, musculoskeletal disorders are associated with impairments in physical, psychological functioning and health-related quality of life [5] and work functioning [6]. Therefore, the burden on quality of life can be substantial in those with hypermobility [7].

Symptomatic hypermobility is more prevalent, but less well-recognised and treated than inflammatory and other arthritides [1], accounting for up to 45% of general rheumatology outpatient referrals (estimated 900,000 attendances in 2014–2015 in England [8]). A quarter of patients attending rheumatology clinics experience anxiety, higher than rates in other medical clinics [9]. In line with this, hypermobility is a risk factor for development [10] and presence of anxiety/panic (OR 4.39, OR 6.72) [11]. This association has long been recognised [10–12]. The health economic impact of hypermobility and anxiety is unknown, but direct cost of anxiety disorders alone in the UK is £11.7 billion [13]. As such, given the associated impairments people with musculoskeletal disorders experience, it is likely experiencing hypermobility and anxiety comorbidly may cost the UK a significant amount of money if adequate treatments are not found and many people with hypermobility may continually struggle with anxiety.

Intervention recommended for generalised anxiety disorder and panic disorder in adults is generally based on the principles of cognitive behavioural therapy (CBT) due to its associated effectiveness [14, 15]. First, people should be educated about these disorders and offered self-help materials and/or psychoeducational groups. Where these do not lead to improvement, formal psychological therapies should be offered: CBT or applied relaxation for generalised anxiety disorder and CBT for panic disorder [15]. Suggestions have been made that CBT may help people with hypermobility develop effective coping strategies, which can be linked to managing chronic pain [16], and work on cognitive distortions [17]. One pilot study used a multidisciplinary rehabilitation programme combining physical and cognitive-behavioural therapy for people with Ehlers-Danlos Syndrome/Joint Hypermobility Syndrome (*n* = 12) and found significant changes in perceived performance of daily activities and self-perceived pain [18], but impact on anxiety was not considered.

To date, no targeted treatments of anxiety exist for people with hypermobility. The brain-body mechanisms underlying this association have been explored in previous research [19] and characterise these by aberrant autonomic control and central representation (i.e. autonomic trait prediction error). These mechanisms are grounded in theoretical models [20, 21] and offer a novel target for a pioneering interventional trial (proof of concept), providing deeper insight into psychophysiological mechanisms of anxiety and its alleviation in hypermobility, helping fill a substantial knowledge and service gap. Data will be used to support future funding bids, with associated impact on patient choice of intervention.

### Choice of comparators

Given the evidence described for treating anxiety with cognitive behavioural principles, ADAPT (Altering Dynamics of Autonomic Processing Therapy) will combine cognitive approaches from clinical health psychology with bio-behavioural training (i.e. interoceptive training based on the work in [20]). This work will target catastrophisation of internal sensations [22] and train participants to more accurately perceive their own heartbeats to reduce *autonomic trait prediction error* (i.e. to increase correspondence between measured changes in heart rate and subjective judgement of these changes) respectively. This active intervention will be compared to a control therapy which replicates therapist contact but is not focussed on interoception or cognitive behavioural approaches, i.e. emotion focussed supportive therapy (EFST). Past evidence suggests that EFST can lead to improvements in anxiety [23].

### Objectives

The primary objective of this study is to assess the efficacy of ADAPT. We hypothesise that participating in ADAPT will lead to significantly reduced anxiety and improvement compared to EFST.

## Method

### Trial design

The design is a randomised controlled trial comparing two non-drug therapies for anxiety in hypermobility (ADAPT vs. EFST). Participants will be blind to the therapy condition they will be in. SPIRIT reporting guidelines [24] have been used throughout this paper.

### Participants

Participants will be recruited from the UK via online advertising and will be 18 years old or over and have lived experience of both joint hypermobility (score ≥ 2 on the hypermobility self-report questionnaire OR a hypermobility related diagnosis: Joint Hypermobility Syndrome [JHS], Hypermobility Spectrum Disorder [HSD], Ehlers-Danlos Syndrome [EDS]) and anxiety (score > 15 on the Beck Anxiety Inventory [BAI]). They will be free of other major psychiatric disorders except comorbid depression. Full inclusion and exclusion criteria are available in Table 1. Stable dose of medication (three months without change) and not currently receiving another form of talking therapy are inclusion criteria adopted to increase the validity of possible findings related to the non-drug therapies used.

**Table 1:** Inclusion and exclusion criteria

	Inclusion criteria	Exclusion criteria
Age	Adults aged 18 years or over	Participants under the age of 18
Capacity	All participants must be able to give informed consent	Unable to give informed consent
Joint hypermobility	Diagnosis of hEDS/HSD/JHS *OR* Score of 2 or more on 5-point questionnaire to detect joint hypermobility	No diagnosis of hEDS/HSD/JHS AND score of 1 or less on 5 point questionnaire to detect joint hypermobility
Anxiety	Self-reported lived experience of anxiety disorder AND A score of 16 or more on Beck Anxiety Inventory endorsing moderate anxiety level AND Anxiety should be the primary psychiatric problem	No self-reported lived experience of anxiety disorder OR A score of 15 or less on Beck Anxiety Inventory OR Anxiety is not the primary psychiatric problem
Other psychiatric disorder	No presence of major psychiatric disorder, except co-morbid depression	Presence of major psychiatric disorder (other than co-morbid depression), e.g. bipolar affective disorder, schizophrenia or psychosis OR personality disorder (e.g. emotionally unstable personality disorder) OR diagnosed neurodevelopmental disorder such as attention deficit hyperactivity disorder or autism spectrum condition OR neurological disorder
Medication use	All participants should be on a stable dose of medication for 3 months OR Medication free AND willing to consider omitting medication that directly affects heart rate (e.g. beta blockers) during the trial	Not on a stable dose of medication (or medication free) for 3 months
Language	All participants must have a reasonable level of both written and spoken English as therapies and assessments will be conducted in English	Poor level of both written and spoken English
Therapy	Not receiving another modality of talking therapy	Currently receiving another modality of talking therapy

The research team will contact interested participants and send them a participant information sheet (PIS). If happy to proceed, a telephone screening will take place to establish if the participant has met the inclusion criteria, and then participants will be referred to a research psychologist to obtain informed consent and conduct the full study assessment measures, in combination with the research assistant (see Table 2 for full list of measures). Those who do not meet the criteria will be informed sensitively and referred on to other services where clinically appropriate. General practitioner (GP) details will be noted during assessment and they will be informed about participation for those eligible.

**Table 2:** Study assessment measures

Questionnaires	Research assistant led	Research psychologist led
Pain and fatigue visual analogue scales [25, 26]	Autonomic assessment	Mini International Neuropsychiatric Interview (MINI [27])
BAI [28]	Interoception assessment [29]	Psychological assessment
Generalised Anxiety Disorder-7 (GAD-7 [30])	Hypermobility assessed using the Brighton Criteria for joint hypermobility syndrome and 2017 Hypermobile Ehlers-Danlos Syndrome (hEDS) Criteria [31]	
Patient Health Questionnaire-9 (PHQ-9 [32])		
Work & Social Adjustment Scale [33]		
Wender Utah Rating Scale [30]		
Ritvo Autism Asperger Diagnostic Scale-Revised [34]		
Anxiety Sensitivity Index [35]		
Toronto Alexithymia Scale-20 [36]		
Dissociative Experiences Scale [37]		
Panic Disorder Severity Scale [38]		
Body Perception Questionnaire [39]		
Autonomic Symptoms of Quality of Life Score [40]		

### Interventions

Two trained clinical psychologists will deliver both intervention arms in order to minimise potential effect of individual therapist. Patients in both intervention groups will receive 8 sessions lasting up to 90 min in duration (12 h total). These sessions will be completed weekly where possible.

#### Emotion-Focused Supportive Therapy (EFST)

EFST is a manualised, non-directive therapy focusing on the emotional experience of the participant and the link emotions may have to events in their life. The intervention focuses on building a safe therapeutic relationship, summarising, labelling and exploring emotions in sessions. Participants can freely choose what is to be covered in each session as it is a non-directive therapy. To control for the confound of homework in the ADAPT intervention arm, participants will also complete weekly homework diaries listing emotional responses to events in the week to be potentially discussed in the subsequent session. As a comparator therapy, the intervention does not include cognitive appraisals or direct participants to the symbiotic relationship between thoughts and behaviours. EFST will not involve any interoception training.

#### Altering Dynamics of Autonomic Processing Therapy (ADAPT)

Expert collaborators recommended combining cognitive approaches from health psychology (targeting catastrophisation of internal sensations [22, 41, 42] with bio-behavioural training to reduce *autonomic trait prediction error* (i.e. to increase correspondence between measured changes in heart rate and subjective judgement of these changes). Cognitive behavioural principles will be used to address anxiety through structured sessions, problem identification, formulation and behavioural experiments. Sessions will focus on addressing beliefs and appraisals around anxiety and safety seeking behaviours, imagery rescripting and homework tasks to be completed between sessions adapted from (Clark, Salkovskis et al. 1999). Participants will be allocated to one of four protocols detailed below (Table 3). Therapists will review homework tasks with participants weekly to assess progress and intervention adherence. Interoception training will be conducted in five of the therapy sessions. Interoception training will be completed via a MATLAB platform on a laptop and will require participants to wear a pulse oximeter (NONIN), which will enable their heartbeats to be recorded. Interoception training will involve two tasks: heartbeat tracking and heartbeat discrimination [29]. In heartbeat tracking, participants will be asked to count their heartbeats across a period of time across 6 trials (range 10–50 s). Participants will start using ‘set B’ with trials in duration between 10 and 25 s. If the participants get 4 trials correct (within 3 beats of the actual answer), they change sets to ‘set A’ with trials 25–50 s in duration. If participants get 4 trials 5 beats or more out on set A, they reduce the next training set back to set B. In heartbeat discrimination, participants will be played their real heartbeat (10 beats); however, the task randomly introduces a delay of 300 ms in some of the trials. Participants will have to decide if the heartbeat they hear is in or out of sync with their actual heartbeat. Participants will complete 20 trials of the heartbeat discrimination task. After each trial, participants will be asked to make a confidence judgement on their response on a visual analogue scale ranging from 0 (not at all confident) to 100 (extremely confident). Participants will be informed after each trial the actual number of heartbeats in the trial or whether the tone was in or out of sync with their actual heartbeat. They will then exercise for 2 min to raise their heart rate and repeat both heartbeat tracking and heartbeat discrimination tasks.

**Table 3:** Therapy protocol session plan

Session	GAD	Panic disorder	Social anxiety	Anxiety NOS
One	◦ Problem identification, goals, formulation, psychoeducation, worry history outcome	◦ Problem identification, goals, formulation, safety seeking behaviours (SSB), psychoeducation, panic diary	◦ Problem identification, goals, formulation, psychoeducation, socialise to video recording, focus of attention work, anxiety trigger diary	◦ Problem identification, goals, formulation, psychoeducation, problem-specific homework diary
	◦ Interoception training	◦ Interoception training	◦ Interoception training	◦ Interoception training
Two	◦ Evidence for beliefs, thought suppression, worry postponement. Worry free zones	◦ Panic diary in formulation, SSB, downward arrow for threat beliefs, selective attention training	◦ Review between session work, identify safety behaviours and avoidance, observer vs. field perspective, attention training	◦ Review homework, socialising to model, belief identification, theory A/theory B
	◦ Interoception training.	◦ Interoception training	◦ Interoception training	◦ Interoception training
Three	◦ Diaphragmatic breathing, challenge beliefs, positive behaviour scheduling	◦ Review formulation, BE relating to feared symptoms, symptom-induction, review BE and beliefs, theory A/theory B	◦ Manipulation of self-focused attention, plan and do BE (video/audio).	◦ Review beliefs and SSB, threat versus coping, plan BE
	◦ Interoception training	◦ Interoception training	◦ Interoception training	◦ Interoception training
Four	◦ Progressive muscle relaxation, attention training, behavioural experiment (BE)	◦ BE, review beliefs, verbal reattribution, theory A/theory B	◦ BE feedback review, prediction versus outcome, explore feared consequences, BE planning	◦ BE, unhelpful thinking styles psychoeducation/survey work
Five	◦ Introduction of the worry tree, imagery re-scripting.	◦ BE, review beliefs	◦ Review BE and beliefs, survey work	◦ Review BE, review goals and formulation, plan BE, imagery re-scripting
	◦ Interoception training	◦ Interoception training	◦ Interoception training	◦ Interoception training
Six	◦ Continue BE, surveys and imagery work continued	◦ BE, review beliefs and imagery	◦ Widening the bandwidth experiments, review BE	◦ Review BE, review goals and formulation, survey/unhelpful thinking styles work
Seven	◦ Continue BE and exploration of beliefs in relation to cognitive formulation	◦ BE, reappraise beliefs, imagery re-scripting	◦ Review BE, imagery re-scripting, function of worry and rumination, BE	◦ Review BE, imagery re-scripting
	◦ Interoception training	◦ Interoception training	◦ Interoception training	◦ Interoception training
Eight	◦ Conclusion of therapy, review goals, relapse prevention	◦ Conclusion of therapy, review goals, relapse prevention	◦ Conclusion of therapy, review goals, relapse prevention	◦ Conclusion of therapy, review goals, relapse prevention

*GAD* generalised anxiety disorder, *BE* behavioural experiment, *NOS* not otherwise specified, *SSB* safety seeking behaviours

### Outcome

Anxiety is the primary outcome, which will be measured by the BAI and group comparisons of mean scores from baseline assessment to end of therapy will be made. Secondary outcomes are as follows: autonomic trait prediction error, interoceptive trait prediction error, presence or absence of psychiatric disorder measured by the MINI [27], the GAD-7 ([43] an additional anxiety measure), depression measured by the PHQ-9 [32], functioning measured by the Work and Social Adjustment Scale [33], anxiety sensitivity quantified by the Anxiety Sensitivity Index [35], alexithymia (i.e. difficulty describing one’s emotions) measured by the Toronto Alexithymia Scale-20 [36], dissociation quantified by the Dissociative Experiences Scale [37] and current panic symptoms measured by the Panic Disorder Severity Scale [38] (see more information on measurement in Table 4). All secondary outcome assessment (except the MINI and feasibility measures) will involve analysing change scores on the measures listed from baseline assessment to end of therapy. The MINI will involve comparing the number of psychiatric disorder presentations from baseline assessment to end of therapy. Trained clinical psychologists will complete psychological interview assessments (including the MINI) and a trained research assistant will complete research assessments.

**Table 4:** Outcome measures

Level of outcome	Outcome measures
Primary outcome	Anxiety levels (BAI) [28]
Secondary outcomes	Autonomic trait prediction error (calculated by z- transforming the mean proportional rise in heart rate on active stand [orthostasis] and subtracting the z-transformed orthostatic sub-scale of the Autonomic Symptoms of Quality of Life Score [40] for each participant)
	Interoceptive trait prediction error (i.e. the mismatch between objective interoceptive accuracy and subjective interoceptive sensibility [44]) will be calculated using the difference between interoceptive sensitivity (i.e. accuracy) and sensibility. Interoceptive sensibility and interoceptive sensitivity scores will be z-transformed and one subtracted from the other. Interoceptive sensitivity score will be calculated across the heartbeat tracking trials at assessment using this equation [44, 45]: $1-\frac{\left|{\mathrm{nbeats}}_{\mathrm{real}}-{\mathrm{nbeats}}_{\mathrm{reported}}\right|}{\left({\mathrm{nbeats}}_{\mathrm{real}}+{\mathrm{nbeats}}_{\mathrm{reported}}/2\right)}$ for heartbeat discrimination trials, this will be calculated by: number of correct trials / number of total trials in assessment Interoceptive sensibility will be assessed using the total score from the Body Perception Questionnaire [39].
	Presence/absence of psychiatric disorder as evidenced by the MINI [27]: Major depressive episode, panic disorder, agoraphobia, social anxiety disorder, obsessive-compulsive disorder, posttraumatic stress disorder and generalised anxiety disorder
	Psychiatric symptomatology as evidenced by scores on the: 1. GAD-7 [43] 2. PHQ-9 [32] 3. Work and Social Adjustment Scale [33] 4. Anxiety Sensitivity Index [35] 5. Toronto Alexithymia Scale-20 [36] 6. Dissociative Experiences Scale [37] 7. Panic Disorder Severity Scale [38]
	Feasibility and acceptability measures of RCT (e.g. practicability, tolerability, Satisfaction with Therapy questionnaire [46])

Feasibility and acceptability of the interventions will be assessed. Qualitative semi-structured interviews, with the research assistant, and completion of the Satisfaction with Therapy questionnaire [46] post-therapy will be completed to assess intervention tolerance, the practicability of the interventions (e.g. including Likert scale questions about whether they found the interoception application simple to use) and tolerance to therapy—questions will include what participants liked and disliked about the intervention they received. Answers in the semi-structured interview and on the questionnaire will be compared across therapy conditions. Further assessment of tolerability will be completed by comparing the number of people who complete therapy and the number of people who withdraw. Reporting of adverse side effects will be compared across conditions as an additional measure of acceptability. Missing data will be quantified (item, instrument and overall assessment level) (which will include participants who withdraw) and used as a measure of acceptability. Completion of homework will be compared across therapy conditions to determine whether there is a difference between how acceptable this appeared in each intervention. Ease of recruitment will be ascertained by calculating the ratio of contacts to the study to the number of participants who are subsequently eligible for and who consent to intervention.

### Sample size

Eighty is the required sample size. Based on previous experimental data (mean anxiety levels in hypermobile subjects BAI = 24.5 [sd 9.36]), this sample size is powered (90% power, 0.05 α) to detect a clinically meaning difference on BAI of 7.5 points (34 participants per group). Thirty-five people per group is the recommended sample size, based on estimating the standard deviation of the primary outcome with good precision [20], to obtain good quality data to inform planning of a future definitive trial. Studies conducted by JAE indicate an attrition rate of approx. 14%; thus, the sample size of 40 per group will take this into account. No further follow-up measures will be obtained if a participant withdraws from the study.

### Recruitment

Participants will primarily be identified through ethically approved advert. Adverts will be displayed at clinical sites and distributed via electronic and paper messaging boards/newsletters to potential participants, e.g. research small ads at University of Sussex and Facebook/Twitter accounts/newsletters of relevant patient/research organisations (e.g. via Brighton and Sussex Medical School [BSMS] and partner universities, by funder (MQ: Transforming Mental Health), and by patient organisations (e.g. Hypermobility Syndromes Association, Ehlers-Danlos Support UK). Electronic advertisement will use geo-targeting to the Sussex and wider South East area where possible. Potential participants will also be identified using the Sussex Partnership NHS Foundation Trust Research Network.

All further contact with potential participants will be via phone, email and post as described below and per potential participant preference.

Interested participants responding to the advert will then contact the research team (email/phone) and be sent (email/post) an appropriate for phase PIS inviting them to contact the research team (email/phone) for further information. A trained member of the research team will then conduct a basic telephone screening to check if potential participants meet inclusion criteria for the study. Participants deemed eligible to take part in the study will subsequently be invited to take part in the study assessment by a study clinical psychologist. A trained research assistant or a member of the research team will collect informed consent from participants prior to undergoing assessment.

The research team will sensitively inform participants if they do not meet the criteria and give an explanation. They will be signposted to relevant support organisations as appropriate and reassured that their routine clinical care will not be affected in any way.

### Treatment allocation and blinding

Participants will be allocated to either ADAPT or EFST through the online randomisation service sealed envelope [47]. Minimisation with a random element (80% probability of being allocated to the arm which minimises imbalance) will be utilised, and participants will be additionally stratified by gender and anxiety score (BAI ≤ 25 vs. > 25). Allocation sequence will not be visible to the psychologists delivering the therapy. Allocation concealment will occur until the participant has been determined eligible for the intervention and sealed envelope reveals to the psychologists which intervention participants have been allocated. Therefore, randomisation will be conducted without any influence of the study team. Psychologists will not be blind to intervention group, due to the nature of the intervention. Whilst they will not reveal to the participant whether the therapy is ADAPT or EFST, due to the nature of the interventions, it is possible participants will not be completely blinded to allocation. A research assistant will be blinded to condition when assessing intervention outcome and questionnaires will be completed without involvement from researchers.

### Data collection

The principal investigator will train the clinical research coordinators, research psychologists and research assistant in data collection, entering, coding and checking as appropriate. Data will be pseudonymised using participant ID. Psychologists will access weekly process data collected prior to therapy sessions starting. The study team will access data using a secure login. Members of the study team will collect interoception data and access this securely. The trained research assistant will assess objective signs of orthostatic intolerance by using a finometer (SMART MEDICAL). Where source data are collected on paper, including the hypermobility (at baseline) and MINI assessments (at baseline and end of intervention), an exact copy of the anonymised data will be manually input into the trial database by a named member of the study team. The research assistant will code all data except treatment allocation group which will be entered by the principal investigator.

Participants will be reimbursed £20 and £25 at the start and end of therapy respectively for completion of trial assessments and to aid retention.

Dropout from the study will be recorded, including reasons where given. Participants are free to withdraw at any point during the study and can request their previously collected data not to be used. Withdrawal or declining to participate will not affect their NHS care in any way; participants are informed of this in the participant information sheet. Participants may be withdrawn from the intervention if their clinical presentation changes in relation to exclusion criteria (e.g. being started on beta-blocker medication, as this could confound the results) or if their clinical condition requires urgent other treatment (e.g. if the participant develops a psychotic disorder). Withdrawn participants will not be replaced.

### Data management

The study team will treat participant information with confidentiality at all times. They will anonymise data held on records and password-protected databases, assign participant ID numbers, and be fully compliant with the General Data Protection Regulation and the Data Protection Act when handling and storing data. Therefore, personal information will be kept separate from the trial data. All members of the research team and other individuals from collaborating trusts or universities involved in collecting, inputting, processing, using and sharing data will have had Information Governance Training. Data management will be a standard item on the agenda for both research team and steering group meetings. Behavioural data will be collected on password protected computers and stored in link-anonymised data files. All data will be backed up to a central storage facility automatically, related to the University of Sussex. Paper files will be kept in locked cabinets.

For statistical analysis, the anonymised data will be downloaded onto password-protected computers. This will be accessible by the trial statistician and principal investigator.

### Statistical methods

Available cases will be analysed following intention to treat principles. The primary outcome will be the BAI which will be compared from baseline assessment to end of therapy. A linear mixed model will be used to test intervention effects, including group comparisons on mean scores of primary and secondary symptom outcome measures. Main effects of condition and time (i.e. repeated measurements) and their interactions will be examined. Effect sizes will be calculated. Missing data will be quantified but no imputation will be performed.

A Data Monitoring Committee has not been arranged, in accordance with the Oxford Clinical Trials Research Unit and the Medicines for Human Use Clinical Trials Regulations (2004); because the intervention occurs over a short period, the protocol will not be modified irrespective of data collected during the intervention and there are minimal risks to participants. No interim analyses are planned.

The independent Trial Steering Committee will help monitor safety (including adverse events), assess adherence to the study protocol and the statistical analysis plan, and oversee progress with data collection. Members include the trial statistician, the PI, the trial consultant clinical psychologist, an independent expert and a patient representative. The principal investigator will regularly conduct data audits to check for possible errors and completeness. If participants or others have concerns regarding the trial conduct, they are advised to contact the Patient Advice and Liaison Service at Sussex Partnership NHS Foundation Trust; this is independent from the investigator and sponsor.

### Patient and public involvement

A Lived Experience Advisory Panel (LEAP) was formed via Sussex Partnership NHS Trust to engage patients and public in design of study, design of materials for ethical approval, and strategies for recruitment and dissemination.

### Adverse events and ethical issues

Adverse events or effects will be monitored throughout the study. It is possible that a participant may evidence risk during the assessment or intervention, such as in relation to reported or likely significant harm to themselves or others. If so, risk will be assessed by the psychologists, discussed with the principal investigator and a supportive plan agreed. This could include informing the participant’s GP, informing appropriate authorities and/or signposting the participant for support where needed. If a serious adverse event occurs, if deemed potentially ‘related’ and/or ‘unexpected’ in relation to the administration of the research procedures, this will be reported to the research ethics committee and sponsor in line with ethical approval. The sponsor (University of Sussex) has appropriate insurance in place.

Research tests conducted are not for diagnostic purposes, and the examination should not be considered an alternative to a proper medical consultation. However, sometimes the joint examination, related assessments (heart rate and blood pressure) or questionnaires may suggest a clinically significant issue. If this is the case or the participant needs further tests, the GP will be contacted in the first instance. The GP will then contact the participant if further tests are required. If the participant has any concerns about this, they are invited to contact a member of research team. The psychologist will make an assessment of whether a further clinical intervention is required at the end of therapy, and, if so, refer to the relevant NHS team for assessment and ongoing management.

To ensure effective dissemination, results will be published in appropriately selected peer-reviewed journals. Summaries of the findings will be published on the BSMS website and links provided to publications. This will aid the dissemination of the research and data, both to other researchers and interested parties. Participants will be sent a summary of the research findings if they consented to this. Fully anonymised data will be made freely available via an open repository, such as Open Science Framework, once the data collection and analyses are completed.

### Changes to method due to COVID-19:

Study assessments will be moved from being completed at the BSMS campus to online assessment (Qualtrics) due to COVID-19 quarantine restrictions. Interoception training will no longer be delivered via MATLAB on computers at BSMS and rather has been developed as an app (HeartRater, CELL SOFTWARE). This app will be installed on a tablet device (Samsung Galaxy Tab A) which will be posted to participants adhering to government safety guidance. Participants will also receive a pulse oximeter (NONIN), which they will wear whilst it is connected to the tablet device via USB connection, to enable their heartbeats to be recorded. Participants will complete both therapies and interoception training in their own homes in a private space on a PC, laptop or smartphone with internet capabilities. Therapy resources will be shared via Zoom videoconferencing software using the secure university account and videocalls will be password protected. All tablet devices will be locked down using Miradore software to ensure participants cannot install interfering software, and the study team can format the tablets remotely if needed. The HeartRater app data will only be accessible to the study team and will require a password to access the online data generated. No personally identifiable information will be stored with the data collected online. Heart rate measurements will not be collected via a finometer and instead will be observed over videoconference using a pulse oximeter sent to participants with the tablet.

## Discussion

This is the first study to test a targeted intervention for people with hypermobility and anxiety. The study will investigate whether ADAPT or EFST are effective in reducing anxiety. The interventions will particularly be compared in relation to anxiety symptoms, but also in terms of autonomic trait prediction error, interoceptive ability, psychiatric disorder, well-being and quality of life measures. While we expect both interventions will be beneficial, the results will help determine their relative effectiveness across the aforementioned outcomes. The trial will also investigate treatment tolerance for remote therapy which has been found to be effective in previous studies in anxiety and depression [48]. An online format has the potential to increase access to and reduce the costs of providing evidence-based intervention to those in need.

## Data Availability

Anonymised datasets and associated material will be available on reasonable request to the corresponding author.
